# Development and validation of an LC–MS/MS method for the quantitation of 30 legacy and emerging per- and polyfluoroalkyl substances (PFASs) in human plasma, including HFPO-DA, DONA, and cC6O4

**DOI:** 10.1007/s00216-021-03762-1

**Published:** 2021-12-15

**Authors:** Gianfranco Frigerio, Simone Cafagna, Elisa Polledri, Rosa Mercadante, Silvia Fustinoni

**Affiliations:** 1 Department of Clinical Sciences and Community Health, University of Milan, via S. Barnaba, 8, 20122 Milan, Italy; 2 Occupational Health Unit, Fondazione IRCCS Ca’ Granda Ospedale Maggiore Policlinico, Milan, Italy

**Keywords:** Per-/polyfluoroalkyl substances, PFAS, LC-MS/MS, Fluorinated alternatives, Emerging PFAS, Per/polyfluoroalkyl acids

## Abstract

Per- and polyfluoroalkyl substances (PFASs) include persistent organic pollutants whose spread is still ubiquitous. Efforts to substitute substances of high concern with fluorinated alternatives, such as HFPO-DA (GenX), DONA (ADONA), and cC6O4, have been made. The aim of this work was to develop and validate an isotopic dilution liquid chromatography-tandem mass spectrometry (LC–MS/MS) method suitable to quantify 30 PFASs in human plasma. Analytes included legacy PFASs (PFOA, PFOS, and PFHxS), fluorinated alternatives (PFBA, PFBS, 6:2 FTSA, HFPO-DA, DONA, and cC6O4), and newly identified compounds (F-53B and PFECHS). The sample preparation was rapid and consisted of simple protein precipitation and centrifugation. Calibration standards and quality control solutions were prepared with a human pooled plasma containing relatively low background levels of the considered analytes. A complete validation was carried out: the lower limits of quantitation (LLOQs) ranged from 0.009 to 0.245 µg/L; suitable linearity (determination coefficients, *R*^2^ 0.989–0.999), precision (2.0–19.5%, relative standard deviation), and accuracy (87.9–113.1% of theoretical) were obtained for considered concentration ranges. No significant variations of analyte responses were recorded under investigated storage conditions and during matrix effect tests. The external verification confirmed the accuracy of the method, although limited to 12 analytes. The method was also applied to 38 human plasma samples to confirm its applicability. The developed assay is suitable for large-scale analyses of a wide range of legacy and emerging PFASs in human plasma. To our knowledge, this is the first published method including cC6O4 for human biomonitoring.

**Graphical abstract:**

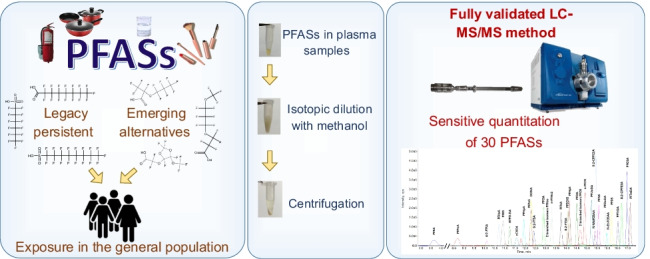

**Supplementary Information:**

The online version contains supplementary material available at 10.1007/s00216-021-03762-1.

## Introduction

Per- and polyfluoroalkyl substances (PFASs) are man-made compounds containing an aliphatic portion characterised by at least one perfluorocarbon moiety. The excellent strength of the carbon–fluorine bond makes perfluorocarbon moieties chemically inert and thermally stable [1]. Despite being an attractive industrial property, perfluorocarbon resistance to degradation raises concerns about environmental fate and human health impact. Indeed, among perfluoroalkyl acid (PFAA) subclasses, perfluoroalkyl-carboxylic and sulfonic acids (PFCAs and PFSAs) count respectively perfluorooctanoic acid (PFOA) and perfluorooctanesulfonic acid (PFOS) as recognised persistent organic pollutants (POPs) under the Stockholm Convention [2]; moreover, perfluorohexanesulfonic acid (PFHxS) is currently a POP candidate [3]. Once released into the environment, PFAAs do not undergo any transformation and are capable of long-range transport; PFAAs are also end products of both biotic and abiotic transformation pathways involving so-called PFAS precursors bearing susceptible functional groups [4]. PFHxS and C_9–14_ PFCA long-chain homologues are also recognised to be persistent and biaccumulative, according to REACH regulation [5–10]. Despite being limited, the above-mentioned long-chain PFAAs are still detected ubiquitously in both environmental [11, 12] and human blood matrices [13, 14]. For these reasons, human body burden assessment of PFASs should be carried out by mainly taking into account PFAAs.

Driven by both voluntary industry initiatives and ever-evolving international regulations, long-chain PFAAs were gradually phased out, including derivates such as perfluorooctanesulfonamide-based compounds [15, 16]. As a result of fluorochemical industry transition, shorter PFAA homologues (e.g. perfluorobutanoic acid, PFBA, and perfluorobutanesulfonic acid, PFBS), partially fluorinated substances (fluorotelomers, e.g. 6:2 fluorotelomersulfonic acid, 6:2 FTSA), and PFASs containing fluorinated carbon chain interspersed with heteroatoms (e.g. per-/polyfluoroalkyl ether acid family, PFEAs) have been conceived, with the aim of replacing traditional compounds with safer alternatives [17].

PFEAs include per-/polyfluoroalkyl ether carboxylic and sulfonic acids (PFECAs and PFESAs) subclasses. Among PFECAs, HFPO-DA (CAS n° 13,252–13-6, also known as GenX), DONA (CAS n° 919,005–14-4, also known as its ammonium salt ADONA), and cC6O4 (CAS n° 1,190,931–41-9) are PFECA substitutes of traditional polymerisation surfactants (as PFOA) [18, 19]. HFPO-DA has been widely encountered in environmental samples [18, 20, 21] and rarely in human biological samples derived from the general population [14], while DONA has only been detected in surface water [22] and in human plasma samples derived from areas impacted by fluorochemical plants [23]. cC6O4 has recently been found in Italy’s largest river basin (Po River, Veneto region, Italy) [24], and it is constantly monitored by regional environmental protection agencies ARPAL [25] and ARPAV [26].

Even though intended as a better option than PFOA, HFPO-DA and PFBS have been recently classified as “substances of very high concern”, according to REACH regulation [27, 28].

The production of perfluoroethylcyclohexane sulfonate (PFECHS) and chlorinated polyfluoroalkyl ether sulfonic acids (e.g. F-53B-related components 6:2 Cl-PFESA and 8:2 Cl-PFESA) is long-standing; nevertheless, their presence has only recently been ascertained in the environment [18, 20, 29–35] and in human biological matrices [36–40]. For these reasons, it is common for authors to refer to these substances as “emerging/alternative”.

Biological monitoring of both legacy and emerging PFASs is a useful approach to carry out integrated and representative assessment of human exposure to these substances. Several methods have been developed for the quantitation of different chemical classes of PFASs in human serum and plasma, by implementing liquid chromatography coupled with tandem mass spectrometry as a suitable analytical technique for high sensitivity and selectivity. Before injection, plasma and serum samples are usually treated to remove proteins [41–45] and/or purified with solid-phase extraction (SPE) [16, 46–58]. However, only a few methods considered the emerging compounds HFPO-DA [16, 43, 44], F-53B [16, 53, 56], DONA [16, 43], or PFECHS [43, 45]. Also, we did not find in the scientific literature methods determining cC6O4 in human blood matrices.

The aim of this work was to develop and validate a high-throughput LC–MS/MS method for the determination of 30 PFASs in human plasma, including both legacy and emerging compounds.

## Materials and methods

### Chemicals

The analytes are reported in Table 1. All analytical standard solutions were purchased from Wellington Laboratories (Guelph, Canada): a mixture containing 24 native standards (PFAC-24PAR), a mixture containing 19 mass-labelled standards (MPFAC-24ES), six separate solutions each containing one emerging PFAS (P5MeODIOXOAc, NaDONA, 9Cl-PF3ONS, 11Cl-PF3OUdS, HFPO-DA, PFECHS), and a solution containing another mass labelled standard (M3HFPO-DA). Names, concentrations, and other specifications of each compound present in these commercial standard solutions are reported in the supplementary material (Table S1 and S2). HPLC-grade acetonitrile, HPLC-grade methanol, and analytical-grade glacial acetic acid were purchased from Sigma-Aldrich (Milan, Italy). Analytical-grade isopropyl alcohol was purchased from Carlo Erba Reagents (Val-de-Reuil, France), analytical-grade 25% ammonia solution was purchased from Merck (Darmstadt, Germany), while purified water was obtained through a Milli-Q Plus ultra-pure system (Millipore, Milford, MA, USA).

**Table 1:**
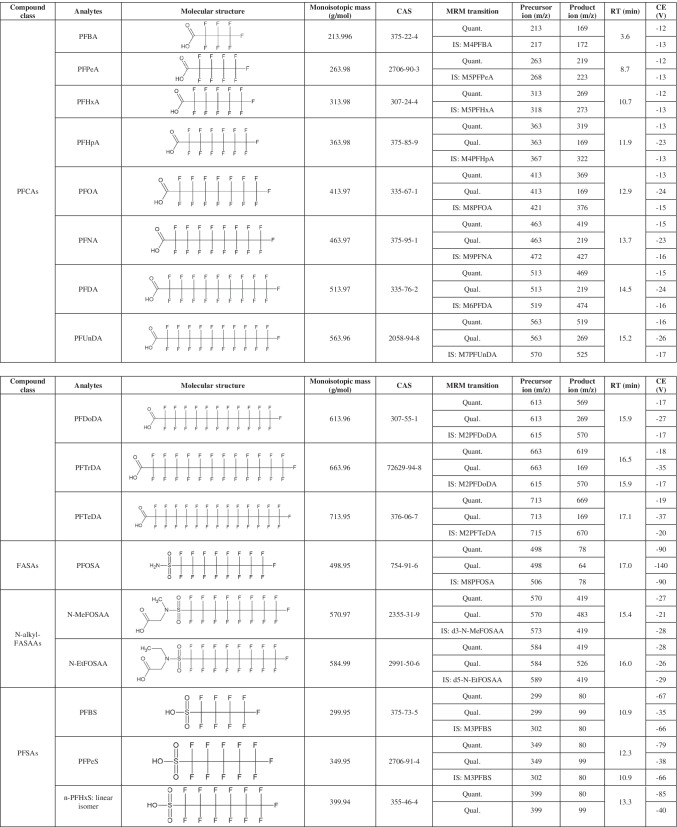

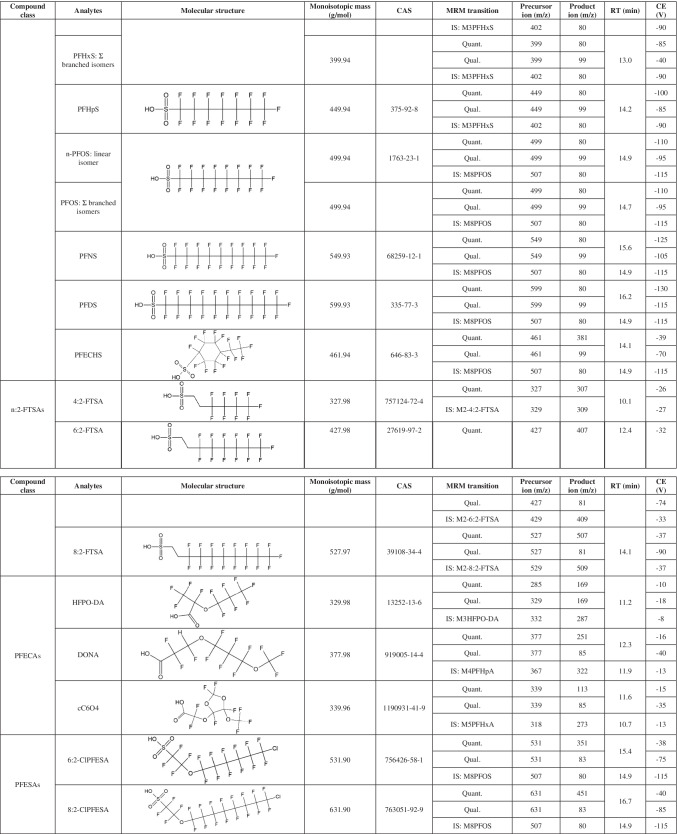
List of the acronyms for analytes, grouped by compound class. For each compound, the molecular structure, monoisotopic mass, Chemical Abstracts Service (CAS) registry number, quantifier (quant.), qualifier (qual.), and internal standard (IS) multiple reaction monitoring (MRM) transitions, chromatographic retention time (RT), and collision energy (CE) are reported. Abbreviations are reported in the homonymous section

### Human plasma samples

Human plasma samples were leftovers obtained from patients of the hospital where the laboratory is located, which had blood collected for other routine clinical measurements. The regulation of the hospital allows the use of routine leftover samples for method development, optimization, and validation as long as they are anonymised. The blood was drawn in EDTA anticoagulant polypropylene tubes and centrifuged at 1127 × g for 15 min to separate plasma, which was transferred to another polypropylene tube. The obtained sample was stored frozen (− 20 °C) until use.

### Blank matrix

Since certain PFASs are widespread in the general population, we analysed several unknown plasma samples and selected the ones characterised by reduced content of the analytes. In particular, the samples with PFOA, PFOS, and PFHxS concentrations up to their first 5^th^ percentile were chosen and mixed to obtain the blank matrix (human pooled plasma). It was stored frozen (− 20 °C) in a polypropylene tube until use.

### Standard solution preparation

Commercial solutions were transferred from their amber glass ampoules to glass vials previously rinsed with methanol (screw cap with PTFE liner), and were finally stored at – 20 °C.

The six standard solutions containing the emerging PFASs were diluted (dilution factor, DF: 25) and mixed in methanol. This solution and the PFAC-24PAR stock solution were diluted with the blank matrix (DF: 50) to obtain the highest calibration standard, which was further diluted with the blank matrix to prepare both the remaining calibration standards and the quality control solutions (QCs). For each analyte, the concentrations ranged from the lower (LLOQ) to the upper limit of quantitation (ULOQ) (Table 2 and Table S3). The method validation was conducted on two QCs per analyte, except for the sum of branched isomers of PFOS, for which high background levels were detected in blank matrix and allowed only one QC level to be included (Table 2 and Table S3).

**Table 2:** Results of method validation. For each analyte, lower and upper limits of quantitation (LLOQ and ULOQ), mean *R*^2^ for investigated linearity range, and within-run precision and accuracy at LLOQ are reported. Within- and between-run precision and accuracy, stability (short term and long term), and matrix effect were calculated at QC levels

	Linearity and sensitivity					Spiked concentration (µg/L)	Precision		Accuracy		Stability		Matrix effect	
Analyte	LLOQ (µg/L)	ULOQ (µg/L)	Mean *R*^2^ (*n* = 6)	Precision at LLOQ (%RDS) (*n* = 5)	Mean accuracy at LLOQ (%theoretical) (*n* = 5)		Within-run precision (%RSD) (min–max) (*n* = 5)	Between-run precision (%RSD) (*n* = 25)	Within-run accuracy (% theoretical) (*n* = 5)	Between-run accuracy (% theoretical) (*n* = 25)	Short-term stability (% variation)	Long-term stability (% variation)	Matrix effect (%RSD) (*n* = 7 plasma samples)	Matrix effect (%theoretical) (*n* = 7 plasma samples)
PFBA	0.078	5.00	0.997	1.8	97.5	0.625	3.9 (1.7–6.5)	5.4	97.7 (89.6–117.5)	98.3	102.2	103.8	10.2	104.5
						2.50	2.7 (1.2–3.7)	3.0	98.5 (91.8–106.4)	99.2	103.1	91.2	4.7	104.9
PFPeA	0.020	1.25	0.998	10.7	116.7	0.078	7.3 (2.8–9.2)	8.8	97.1 (89.6–109.7)	100.3	99.4	105.1	6.6	110.7
						0.625	3.8 (1.7–5.9)	4.3	97.8 (93.2–101.1)	98.9	100.4	96.8	6.1	108.8
PFHxA	0.039	2.50	0.996	8.3	104.2	0.078	7.6 (4.1–11.5)	9.2	96.0 (87.3–104.6)	99.0	96.8	100.6	8.8	104.2
						0.625	3.2 (1.6–4.1)	3.4	97.4 (92.1–103.7)	98.2	99.2	96.6	4.9	111.7
PFHpA	0.039	2.50	0.997	16.2	101.4	0.078	12.6 (8.7–15.8)	12.3	87.9 (59.2–106.9)	93.6	94.7	105.0	18.7	98.8
						0.625	4.2 (2.3–7.2)	5.2	97.7 (87.2–110.1)	99.1	95.4	104.9	6.6	105.6
PFOA	0.156	40.0	0.997	17.9	101.7	0.625	11.4 (6.0–16.9)	11.2	95.9 (67.7–118.3)	95.4	105.5	87.4	18.9	107.5
						2.50	4.2 (3.3–6.3)	4.0	94.8 (86.5–104.4)	95.6	98.7	102.3	7.7	105.5
PFNA	0.078	5.00	0.996	12.2	119.5	0.625	7.3 (2.7–9.4)	7.4	96.6 (82.6–107.7)	95.9	95.8	100.1	5.1	103.4
						2.50	4.2 (3.2–5.2)	4.5	97.6 (88.5–108.5)	97.7	104.1	97.3	5.5	104.0
PFDA	0.078	5.00	0.997	14.3	114.0	0.625	7.1 (3.7–11.9)	7.5	97.4 (85.9–106.1)	99.0	94.6	98.7	7.8	104.3
						2.50	5.6 (5.1–6.4)	5.8	97.8 (87.7–106.8)	98.1	106.1	88.8	5.5	108.7
PFUnDA	0.078	5.00	0.998	9.8	99.2	0.625	4.7 (1.8–7.4)	5.5	96.8 (88.6–104.7)	98.5	97.6	95.7	8.9	105.4
						2.50	5.7 (3.3–7.7)	6.1	95.0 (89.0–102.0)	97.7	100.0	94.2	6.2	113.0
PFDoDA	0.039	2.50	0.997	9.0	105.3	0.078	6.4 (3.5–11.2)	7.4	96.0 (76.9–110.0)	97.1	99.9	111.6	9.8	102.9
						0.625	5.1 (3.2–8.0)	5.4	98.2 (87.7–107.7)	100.3	99.7	110.3	6.4	110.7
PFTrDA	0.020	1.25	0.997	15.8	103.1	0.078	6.4 (4.0–7.6)	13.7	98.5 (80.6–116.1)	99.5	105.8	87.4	11.1	103.5
						0.625	6.4 (4.2–9.1)	11.1	100.0 (89.2–111.2)	101.4	108.7	87.4	8.1	113.3
PFTeDA	0.020	1.25	0.999	8.1	97.6	0.078	5.0 (3.3–7.3)	5.2	96.8 (85.5–110.2)	98.7	97.4	100.0	10.3	99.7
						0.625	2.7 (1.2–4.6)	3.4	100.8 (93.5–108.1)	101.0	102.0	102.0	5.4	112.1
PFOSA	0.010	1.25	0.998	9.3	117.0	0.078	3.8 (3.1–4.8)	3.9	94.8 (84.7–105.4)	97.3	96.5	105.2	8.3	100.0
						0.625	3.5 (2.3–6.0)	3.9	96.7 (88.3–100.5)	98.6	101.3	99.4	4.8	109.1
N-MeFOSAA	0.039	2.50	0.997	7.7	107.8	0.078	12.9 (7.8–20.0)	15.1	98.8 (82.2–123.9)	99.7	89.1	93.8	19.2	97.1
						0.625	7.6 (4.6–12.2)	8.2	99.7 (88.2–114.6)	100.3	99.6	95.9	7.5	102.7
N-EtFOSAA	0.039	2.50	0.997	18.6	107.1	0.078	14.3 (6.4–20.6)	14.8	103.7 (77.8–157.3)	101.2	108.8	106.6	10.3	91.6
						0.625	6.5 (4.8–8.9)	6.8	101.7 (87.6–121.2)	102.7	105	107.6	4.8	106.9
PFBS	0.035	2.22	0.998	13.9	90.8	0.069	5.6 (4.5–6.3)	6.5	101.7 (80.5–136.3)	95.3	113.5	96.3	13.4	105.4
						0.555	5.9 (4.6–7.5)	6.1	102.8 (95.0–109.9)	101.9	102.6	92.1	6.6	112.6
PFPeS	0.018	1.18	0.998	12.6	114.3	0.074	8.6 (4.1–11.8)	9.2	102.0 (82.0–128.3)	103.0	105.9	113.0	19.7	90.5
						0.589	7.5 (2.0–10.7)	8.2	98.4 (78.3–108.9)	102.5	87.4	97.6	19.2	106.9
n-PFHxS	0.116	7.42	0.995	7.0	101.0	0.464	4.6 (1.8–7.0)	5.6	101.5 (74.5–114.7)	97.7	104.6	111.0	10.2	98.6
						1.85	4.3 (2.4–5.2)	4.4	98.3 (87.1–115.7)	98.5	102.4	100.4	7.8	103.2
PFHxS: Ʃ branched isomers	0.013	0.862	0.996	14.5	93.0	0.108	9.5 (4.9–16.0)	11.9	100.0 (82.7–124.1)	97.6	104.1	92.2	8.7	104.0
						0.431	5.3 (3.3–7.8)	9.1	96.5 (83.2–108.7)	96.8	105.6	98.5	9.5	103.1
PFHpS	0.074	4.76	0.997	8.3	113.1	0.595	6.8 (4.8–9.9)	9.1	98.1 (87.8–111.4)	98.6	99.1	87.8	18.8	108.0
						2.38	4.8 (2.1–6.5)	7.9	98.5 (87.8–109.3)	99.3	108.0	85.5	18.9	105.6
n-PFOS	0.229	29.3	0.997	14.2	101.6	0.457	15.4 (10.0–20.3)	16.3	113.1 (76.8–152.5)	107.2	108.2	89.9	7.7	91.6
						1.829	6.8 (4.3–10.3)	6.9	95.8 (81.5–109.8)	96.5	104.1	104.8	8.1	102.2
PFOS: Ʃ branched isomers	0.245	7.84	0.997	10.8	99.9	0.490	8.9 (6.1–14.4)	9.0	92.6 (73.5–112.7)	97.1	100.4	104.8	13.5	98.3
PFNS	0.038	2.40	0.997	5.0	107.8	0.075	6.7 (4.3–7.5)	6.8	95.5 (82.6–111.5)	99.9	96.2	114.5	14.1	104.9
						0.601	3.4 (1.2–5.5)	3.9	98.2 (88.1–109.1)	100.2	99.4	105.7	11.1	105.3
PFDS	0.019	1.21	0.997	7.2	98.5	0.076	12.1 (7.9–16.7)	17.6	96.1 (80.6–118.1)	98.2	97.2	110.9	8.6	110.1
						0.604	6.4 (3.7–9.2)	10.0	101.7 (92.6–113.1)	103.4	97.8	102.8	7.6	111.3
PFECHS	0.009	1.16	0.993	16.0	95.8	0.072	15.7 (7.8–21.2)	17.5	103.4 (82.8–133.0)	101.8	95.8	87.9	9.7	94.7
						0.578	7.3 (4.2–14.7)	11.5	97.9 (88.5–109.8)	98.2	109.5	90.3	9.9	101.9
4:2 FTSA	0.018	1.17	0.997	13.3	95.4	0.073	9.4 (7.2–12.3)	9.3	97.7 (82.1–115.2)	98.8	99.6	95.5	8.4	109.0
						0.586	6.4 (2.5–10.5)	8.6	103.0 (94.5–113.4)	100.1	108.2	101.0	6.5	112.1
6:2 FTSA	0.019	1.19	0.995	16.1	112.4	0.074	10.2 (9.4–10.7)	9.8	99.2 (83.1–127.8)	100.9	95.6	111.5	15.0	110.6
						0.595	5.1 (3.3–7.4)	5.2	101.5 (89.0–114.4)	102.5	99.1	94.1	9.1	111.8
8:2 FTSA	0.038	1.20	0.994	18.9	96.6	0.075	12.7 (10.3–15.9)	14.1	100.2 (80.0–131.7)	100.2	99.4	104.6	9.0	102.2
						0.601	4.9 (3.3–6.3)	5.4	103.0 (84.5–115.6)	100.7	103.2	95.5	8.6	106.3
HFPO-DA	0.020	1.25	0.989	15.2	95.3	0.078	8.7 (6.5–11.0)	9.0	94.0 (82.7–109.1)	97.2	95.9	84.9	14.7	101.0
						0.625	6.7 (2.0–9.7)	7.4	97.8 (72.7–131.9)	99.8	88.8	112.5	16.8	112.0
DONA	0.018	1.18	0.996	15.3	101.5	0.074	8.6 (5.4–11.5)	8.5	91.8 (78.0–104.5)	102.2	92.6	90.3	9.4	97.4
						0.590	4.1 (2.5–7.1)	5.3	94.6 (84.8–107.6)	103.6	94.2	89.1	9.7	104.6
cC6O4	0.020	1.25	0.994	15.4	90.9	0.078	8.3 (4.1–11.5)	8.3	94.0 (76.5–134.0)	96.6	97.8	111.3	9.4	97.3
						0.625	7.8 (3.5–9.9)	10.3	97.3 (85.0–108.2)	98.4	106.9	97.5	9.3	97.5
6:2 Cl-PFESA	0.018	1.17	0.997	18.9	103.9	0.073	6.5 (3.2–10.0)	9.2	99.6 (85.9–119.5)	99.0	106.1	96.4	13.1	92.0
						0.584	6.6 (4.7–9.0)	9.2	102.9 (91.0–117.8)	100.6	111.1	100.1	10.7	103.3
8:2 Cl-PFESA	0.009	1.18	0.997	10.6	96.8	0.074	7.1 (5.2–10.0)	8.6	101.0 (83.3–117.0)	101.9	104.5	107.6	10.5	96.3
						0.590	4.7 (1.8–8.7)	6.4	103.9 (95.2–113.9)	102.9	110.3	104.9	10.9	102.9

An internal standard working solution, containing the 20 isotopic labelled standards, was prepared in methanol by diluting and mixing M3HFPO-DA (DF: 25,000) and MPFAC-24ES (DF: 2500).

All prepared standard solutions were stored (− 20 °C) in 2.0-mL glass vials (screw cap with PTFE liner) until use.

### Sample preparation

An aliquot of 20 µL of plasma sample was dispensed into polypropylene conical tubes, and 80 µL of the internal standard working solution was added. Since the internal standard working solution was prepared in methanol, this step was also aimed to crash plasma proteins. The obtained solution was thoroughly vortexed and centrifuged at 10,500 × g for 15 min. The supernatant was then collected and transferred in an autosampler vial (screw cap with self-sealing PTFE septa) containing a 250-µL polypropylene insert. The vial was finally placed in the thermostated autosampler until analysis. The same procedure was followed for unknown samples, sample blanks (unspiked blank matrix), procedural blanks (methanol treated as an unknown sample), calibration standard solutions, and QCs.

### LC–MS/MS analysis

The LC–MS/MS system consisted of an Agilent 1260 liquid chromatograph (Agilent Technologies, Cernusco sul Naviglio, Italy) coupled with a Q-Trap 5500 mass spectrometer (AB Sciex, Monza, Italy) equipped with an electrospray ionisation source (ESI). The analytical column used was an Acquity HSS T3 C18 (2.1 × 100 mm, 1.8 µm) (Waters, Sesto San Giovanni, Italy), with a guard-column SecurityGuard C18 (4 × 3 mm) (Phenomenex, Castel Maggiore, Italy), both installed in a column compartment kept at 45 °C ± 1 °C. A Hypersil GOLD column (3 × 50 mm, 3 µm) (Thermo Fisher Scientific, Rodano, Italy) was installed between the pump and the autosampler injector in order to delay PFAS contaminations deriving from mobile phases. The autosampler temperature was set at 10 °C, and the injection volume was 10 µL. Between each sample withdrawal and injection, the autosampler syringe was flushed for 5 s with a solution composed of water, methanol, and isopropyl alcohol (2:1:1 v/v). The chromatographic run consisted of a linear gradient at the constant flow rate of 0.2 mL/min. Mobile phase A was composed of an aqueous solution of 10 mM ammonium acetate with 0.1% acetic acid (pH 4–4.5), while mobile phase B was acetonitrile. The HPLC gradient programme was optimised as follows: the initial percentage of B was 20%, then it increased from 20 to 40% (0–0.5 min); was kept constant at 40% (0.5–1.5 min); increased from 40 to 100% (1.5–11.5 min); was kept in isocratic condition at 100% (11.5–18.5 min); then decreased from 100 to 20% (18.5–18.6 min); and finally was maintained at 20% (18.6–28.6 min, this latter step conditioned the column for the following analysis). A programmed valve diverted the flow from the analytical column to waste during the first 2 min, between 5 to 7 min, and after 18 min up to the end of the HPLC method: this configuration minimised in-source contamination when the eluent contains no analytes. The mass spectrometer operated in negative polarity with a scheduled multiple reaction monitoring scan type (s-MRM), with retention time acquisition windows of 180 s and a target cycle time of 1 s. The transitions used for detecting the analytes, the declustering potential, the collision energy (CE), and the collision exit potential were optimised with a manual tuning through direct infusion of diluted standard solutions; precursor/product ion pairs and CE are reported in Table 1. For each analyte, the precursor ion corresponded to the deprotonated molecular ion [M − H]^−^, except for HFPO-DA which underwent an in-source fragmentation. We monitored two transitions for each native standard (when available): the most intense was suitable for the quantitation of the analyte in matrix (quantifier, quant.), while the second one was used as a confirmation (qualifier, qual.). For each internal standard, the most intense transition was recorded, coinciding with the corresponding analogue quant., except for M3HFPO-DA. Since not all analogue internal standards of considered native analytes were commercially available, the remaining analytes were paired with structurally similar mass labelled standards (Table 1). Other general mass spectrometer parameters were manually optimised by flow-injection of diluted standard solutions: in particular, curtain gas (nitrogen) was set to 35 psi, ion spray voltage − 2500 V, turbo heater temperature 450 °C, gas 1 (air) pressure 60 psi, gas 2 (air) pressure 30 psi, and collision gas (nitrogen) was set to “high”. The Analyst® software was used to prepare acquisition method and analytical batches (version 1.7.1, AB Sciex, Monza, Italy), while Multiquant™ (version 3.0.31721.0, AB Sciex, Monza, Italy) was used for data elaboration. The analyte response (area ratio) was recorded as the ratio between the peak area of the native standard and the peak area of the assigned internal standard.

### Method validation

The method was thoroughly validated following the guidelines reported by the Food and Drug Administration (FDA) [59], the Italian Society of Clinical Biochemistry and Clinical Molecular Biology (SIBioC) [60], and the International Council for Harmonisation of Technical Requirements for Pharmaceuticals for Human Use (ICH) [61].

### Linearity

The standard solutions used for calibration curves were prepared as reported in the “Standard solution preparation” section. Fourteen calibration standard solutions, covering a wide range of concentrations, were injected twice within each analytical sequence, along with a repetition of six replicates of blank matrix. For each analyte, the calibration curve consisted of at least six non-zero calibrators and was calculated by plotting the blank subtracted area ratios as *y*-values and the known concentrations (µg/L) as *x*-values; a 1/*x* weighted least-squares linear regression was computed. Linearity was assayed by calculating the mean coefficient of determination, *R*^2^: three independent calibration curves were prepared and analysed separately in three analytical batches over the course of 6 months. The acceptance criteria followed to ensure the quality and reproducibility of each calibration curve are those described in the “calibration curve” section of the FDA guidelines. The use of blank subtracted calibration curve was in agreement with ICH guideline M10 on bioanalytical method validation [61].

### Selectivity and carryover effect

In order to evaluate the selectivity of the method, procedural blanks (unspiked methanol prepared as an unknown sample) and blank samples (unspiked blank matrix) were analysed both with and without adding the internal standard solution; solvent blanks (pure methanol) were also analysed. The presence of interfering peaks with the same retention time of quant. transitions or internal standard transitions was verified. Moreover, the interference from internal standards was evaluated by comparing the quant. chromatograms obtained by analysing replicates of pooled plasma samples with those obtained by analysing replicates of pooled plasma samples prepared without internal standards.

To evaluate the carryover effect, two analyses of the solvent blanks were carried out right after every analysis of the highest calibration standard level.

### Sensitivity

For each analyte, the lower limit of quantitation (LLOQ) was calculated using the following formula: LLOQ = (10SEq + *q*)/*m*, where *q* is the intercept of the calibration curve (calculated only if positive), *m* is the slope, and SEq is the standard error of *q* [62]; if the intercept was negative, the formula was reduced to 10SEq/*m*. LLOQ was obtained as a mean from three independent calibration curves prepared and analysed separately in three analytical batches over the course of 1 month. Within-run precision and accuracy (“Precision and accuracy” section) at LLOQ concentrations were further experimentally determined by analysing five independent replicates of spiked blank matrix: for each analyte, the LLOQ was confirmed if the mean accuracy was within ± 20% of the theoretical value, and the RSD % was ≤ 20%.

### Precision and accuracy

To test the method precision, the preparation and analysis of each QC sample were repeated five times per run (within-run), and for five different runs over the course of 2 weeks (between-run). Relative standard deviations (%RSDs) of the blank subtracted area ratios were calculated among the analyses carried out within each run; the within-run precision was calculated as the mean of these %RSDs. The between-run precision was calculated as the %RSD among all the analyses.

To test the accuracy of the method, the preparation and analysis of each QC sample were repeated five times per run (within-run), and for five different runs over the course of 8 months (between-run). Accuracy was calculated by dividing the calculated concentrations in the spiked samples by the theoretical spiked concentration and multiplying by 100 (% theoretical). For each analyte, the within-run accuracy was calculated as the average of the mean accuracies obtained within each analytical batch, while the between-run accuracy was calculated as the mean accuracy obtained from all analyses.

### Stability

Short-term stability was tested to verify the stability of the prepared samples while stored at 10 °C in the autosampler: two replicates of QC samples were analysed right after preparation and following 1 week of storage. The short-term stability was calculated as the % ratio between the area ratios obtained from the analyses of the stored QCs and those obtained by analysing freshly prepared QCs.

Long-term stability was tested to verify the stability of analytes in the matrix, from the sample collection to the analytical measurement while kept at – 20 °C. Blank matrix was spiked with the native standard solutions at the concentration of QCs; then, for each level, an aliquot was immediately prepared and analysed, along with the calibration curve, while another aliquot was frozen at – 20 °C. After 1 month, the second aliquot was defrosted at room temperature, prepared, and analysed along with a freshly prepared calibration curve. Long-term stability was calculated as the % ratio between calculated concentrations of the stored QC samples and those obtained with freshly prepared QCs.

### Matrix effect

Seven plasma samples (previously screened for relatively low background levels of analytes), each derived from different individuals, were spiked with the native standards at QC concentrations, in duplicate. For each analyte, the area ratio was subtracted by the area ratio obtained in the corresponding non-spiked sample, and the results were compared among the seven different samples. Between-sample precision and accuracy were determined at each QC level.

### External verification

The accuracy of the method was further verified for 12 analytes (PFPeA, PFHxA, PFHpA, PFOA, PFNA, PFDA, PFUnDA, PFDoDA, PFBS, PFHxS, PFHpS, and PFOS), through the analyses of serum samples which had been prepared in the frame of the interlaboratory comparison investigations and external quality assurance schemes (ICI-EQUAS) carried out during the HBM4EU project [63–65]. We did not participate in this exercise, but we used these samples as reference standard material. The sera were stored frozen (− 20 °C) until use. For each ICI-EQUAS round, two levels (low and high) of considered PFASs were available; the samples from three different rounds (2, 3, and 4) were analysed in two independent analytical sequences 6 months apart from each other. Accuracy and *Z*-score were calculated to compare our results to reference values reported in the HBM4EU final reports. Mean *Z*-scores were calculated using the following expression: *Z* =|(*x* − *C*) / *σT*|, where *x* is our calculated concentration, *C* is the reference concentration, and *σT* is a fit-for-purpose targeted standard deviation calculated as 0.25*C. According to the ICI-Equas guidelines, *Z*-scores ≤ 2 are considered satisfactory [66].

Furthermore, we participated in the round 67 of the German External Quality Assessment Scheme (G-EQUAS) for the external verification of PFOA and PFOS. In this case, two serum samples were analysed in blind; the results were submitted and then compared to those obtained from reference laboratories [67, 68].

### Analytical sequence

For routine measurements, a typical sequence consisted of a few injections of pure methanol, followed by blank samples, all the fourteen calibration standard solutions, QCs, a set of unknown samples interspersed by repetitions of QCs, and finally a second injection of the calibration standard solutions. Each sequence was considered acceptable if at least 75% and a minimum of six non-zero calibrator levels were within ± 15% of their theoretical concentrations, except at LLOQ, for which an inaccuracy up to ± 20% was accepted, and if at least 67% of all QC samples were within ± 15% of their theoretical values, with at least 50% of QC samples per level were within ± 15% of their theoretical values [59].

### Method application and statistical analyses

The developed method was applied to 38 plasma samples collected from the local general adult population (see “Human plasma samples” section for details). Non-quantifiable values were replaced with half of the LLOQ, then descriptive statistics was applied (median, 5^th^ and 95^th^ percentiles). Statistical analyses were performed using the R software (version 4.0.5) [69], with the Rstudio interface (Version 1.4.1106 RStudio, PBC, Boston, MA, USA), and the tidyverse package [70].

## Results

### Method validation

**Fig. 1:**
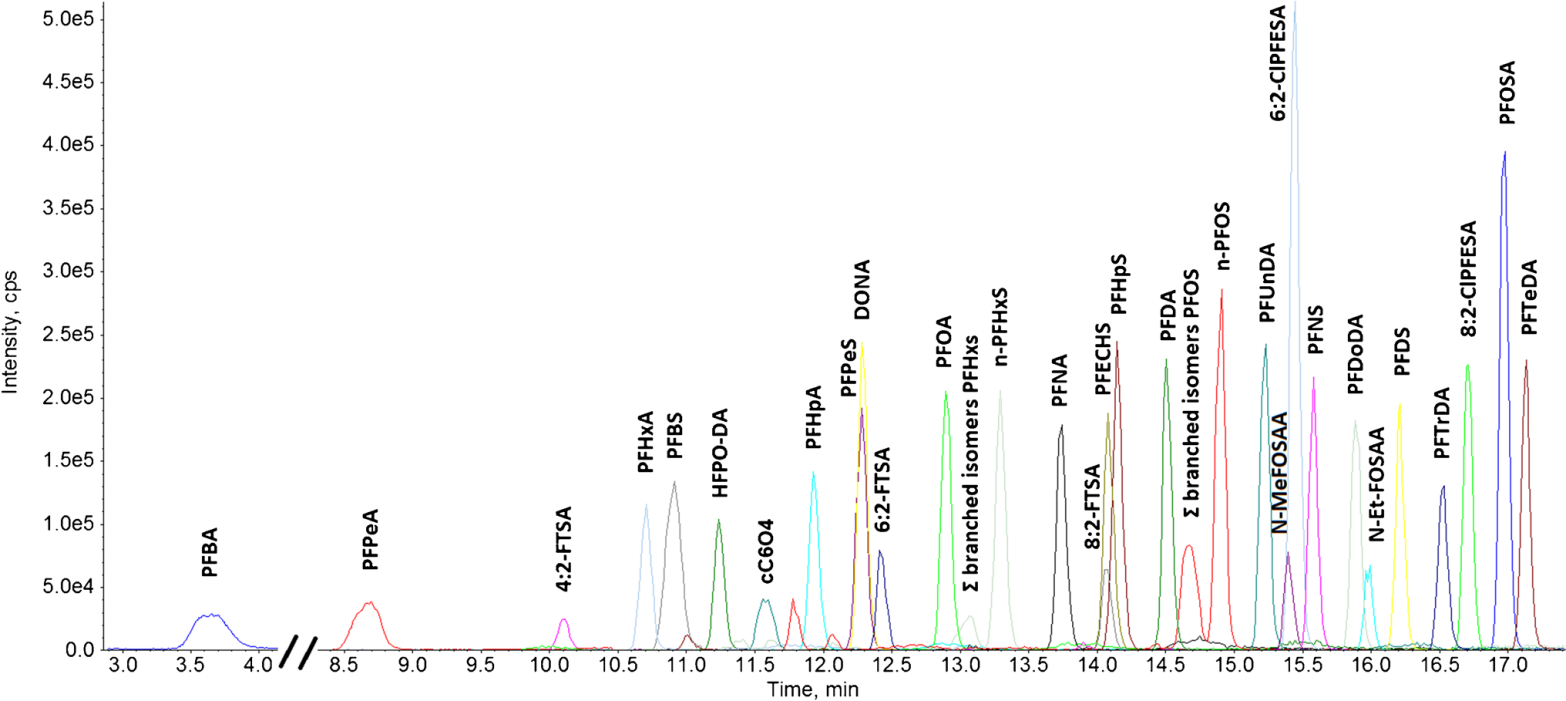
Superimposed extracted ion chromatogram of quantifier transitions obtained from an analysis of a pooled plasma sample spiked with the analytical standards at the concentrations of the level 10 (complete concentrations are reported in Table S3)

Figure 1 shows the extracted ion chromatograms of quant. transitions obtained from an analysis of blank sample spiked with the analytical standards. The analytes are separated and eluted in 18 min. For both PFHxS and PFOS, the linear isomer was separated from the branched isomers, which were independently quantified as sum of all the possible branched isomers.

### Linearity

The mean *R*^2^ of each analyte ranged from 0.989 to 0.999 (Table 2), thus showing a good linearity for the considered concentration ranges.

### Selectivity and carryover effect

As expected, all human plasma samples analysed contained many PFASs, some of which were detected at trace levels and others at significant concentrations (PFOA, PFOS, and PFHxS). The background concentration of each analyte found in the blank matrix used for method validation is reported in Table S4. Nonetheless, no interfering peaks having the same retention time of internal standard and quantifier transitions were detected, except for a co-eluting peak from the matrix at a close retention time of the quant. transition of PFBS that affected its sensitivity. Zero calibrators were free of interference at the retention time of the internal standards, according to FDA guidelines; the contribution of internal standards to the peak area of quant. transitions was not significant.

Over the course of 1 year, no significant carryover effect was observed as no considerable peaks in solvent blanks were observed, according to FDA guidelines.

### Sensitivity

We found a good match between theoretically calculated LLOQ and the experimental verification. Indeed, the precision and the accuracy at LLOQ ranged respectively from 1.8 to 18.9% (%RSD), and from 90.8 to 119.5% (%theoretical) (Table 2). LLOQ values ranged from 0.009 to 0.078 µg/L for most compounds, with the exception of PFOA (0.156 µg/L), n-PFHxS (0.116 µg/L), n-PFOS (0.229 µg/L), and PFOS ∑ branched isomers (0.245 µg/L) for which we observed the highest background levels in blank matrixes. However, these levels were still suitable for an adequate quantitation of these compounds in samples from the general population (see “Method application” section).

### Precision and accuracy

The results of the within- and between-run accuracy and precision tests are reported in Table 2. The within-run mean %RSD of blank subtracted area ratios ranged from 2.7 to 15.7%, while overall between-run %RSD ranged from 3.0 to 17.6%. Within-run mean accuracy ranged from 87.9 to 113.1%, while between-run accuracy ranged from 93.6 to 107.2%. The analyses were performed over the course of 8 months, thus showing the robustness of the method.

### Stability

The results of the short-term stability (prepared samples stored for 1 week at 10 °C in the autosampler) ranged from 87.4 to 113.5%, while the results of the long-term stability (QC samples stored at – 20 °C for 1 month) ranged from 84.9 to 114.5% (Table 2), thus showing no significant alterations of analyte responses over time.

### Matrix effect

The matrix effect, calculated as %RSD of the blank subtracted area ratios among seven different plasma samples, ranged from 4.7 to 19.7%, while the calculated concentrations ranged from 90.5 to 113.3% of the theoretical values (Table 2).

### External verification

**Fig. 2:**
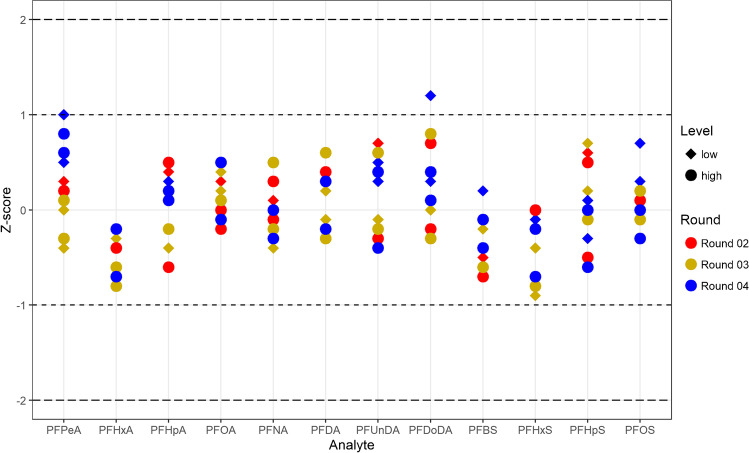
Results obtained from the analyses of samples from the interlaboratory comparison ICI-EQUAS. We did not participate in the exercise, but, using these samples as reference material, we compared our results with the reported reference values. *Z*-scores are plotted for each of the 12 analytes included in the exercise: the samples from three different rounds were analysed in two independent analytical sequences 6 months apart from each other

The results of the analyses of the ICI-Equas samples are presented in Fig. 2 and reported in the supplementary material (Table S5). If compared with reference values established by expert laboratories, mean accuracy ranged from 82.1 to 119.2% and the mean *Z*-score ranged from 0.1 to 0.8. The participation in the G-Equas round 67 for PFOA and PFOS was evaluated as satisfactory (Table S6 and Fig. S1).

### Method application

The results of the method application to 38 plasma samples are reported in Table 3. PFOA, PFNA, PFDA, n-PFHxS, n-PFOS, PFOS Ʃ branched isomers, and PFECHS were always detected (≥ LLOQ). We obtained high detection frequencies (≥ 70% and < 100%) for other long-chain PFCAs (PFUnDA and PFTrDA) and some PFSA homologues (PFPeS, PFHxS Ʃ branched isomers, and PFHpS), while the short-chain perfluoroalkyl acids PFBA and PFBS were found in few samples. PFOA, n-PFOS, and PFOS Ʃ branched isomers showed the highest median levels (1.497, 1.909, and 1.267 µg/L, respectively), followed by n-PFHxS (0.580 µg/L) and C_9–11_ PFCAs homologues. The emerging analytes, HFPO-DA, DONA, and cC6O4, were mostly not quantifiable in the considered samples.

**Table 3:** Results of the application of the method to the plasma samples (*n* = 38). Data are reported as median and 5^th^ and 95^th^ percentiles, along with the number (and percentage) of samples with concentrations greater than or equal to the lower limit of quantitation

Analyte	5th percentile (µg/L)	Median (µg/L)	95th percentile (µg/L)	Samples ≥ LLOQ (percentage)
PFBA	< LLOQ	< LLOQ	0.104	3 (8%)
PFPeA	< LLOQ	< LLOQ	< LLOQ	0 (0%)
PFHxA	< LLOQ	< LLOQ	< LLOQ	0 (0%)
PFHpA	< LLOQ	< LLOQ	0.194	18 (47%)
PFOA	0.952	1.497	3.565	38 (100%)
PFNA	0.274	0.409	0.748	38 (100%)
PFDA	0.106	0.198	0.329	38 (100%)
PFUnDA	< LLOQ	0.139	0.310	29 (76%)
PFDoDA	< LLOQ	< LLOQ	0.058	5 (13%)
PFTrDA	< LLOQ	0.031	0.073	27 (71%)
PFTeDA	< LLOQ	<LLOQ	< LLOQ	1 (3%)
PFOSA	< LLOQ	< LLOQ	< LLOQ	1 (3%)
N-MeFOSAA	< LLOQ	< LLOQ	0.050	3 (8%)
N-EtFOSAA	< LLOQ	< LLOQ	< LLOQ	1 (3%)
PFBS	< LLOQ	< LLOQ	0.086	10 (26%)
PFPeS	< LLOQ	0.029	0.076	28 (74%)
n-PFHxS: linear isomer	0.275	0.580	1.692	38 (100%)
PFHxS: Ʃ branched isomers	< LLOQ	0.035	0.079	35 (92%)
PFHpS	< LLOQ	0.093	0.249	29 (76%)
n-PFOS: linear isomer	1.113	1.909	4.679	38 (100%)
PFOS: Ʃ branched isomers	0.612	1.267	3.279	38 (100%)
PFNS	< LLOQ	<L LOQ	< LLOQ	0 (0%)
PFDS	< LLOQ	< LLOQ	< LLOQ	0 (0%)
PFECHS	0.021	0.041	0.135	38 (100%)
4:2-FTSA	< LLOQ	< LLOQ	< LOQ	0 (0%)
6:2 FTSA	< LLOQ	< LLOQ	< LOQ	1 (3%)
8:2 FTSA	< LLOQ	< LLOQ	< LOQ	1 (3%)
HFPO-DA	< LLOQ	< LLOQ	< LOQ	0 (0%)
DONA	< LLOQ	< LLOQ	< LOQ	0 (0%)
cC6O4	< LLOQ	< LLOQ	< LOQ	1 (3%)
6:2 Cl-PFESA	< LLOQ	< LLOQ	0.020	6 (16%)
8:2 Cl-PFESA	< LLOQ	< LLOQ	< LùLOQ	0 (0%)

For calculation of the median and 5^th^ and 95^th^ percentiles, the non-quantifiable values were replaced with half of the LLOQ. Replacement values were the following: PFBA: 0.039; PFPeA: 0.010; PFHxA: 0.020; PFHpA: 0.020; PFOA: 0.078; PFNA: 0.039; PFDA: 0.039; PFUnDA: 0.039; PFDoDA: 0.020; PFTrDA: 0.010; PFTeDA: 0.010; PFOSA: 0.005; N-MeFOSAA: 0.020; N-EtFOSAA: 0.020; PFBS: 0.018; PFPeS: 0.009; n-PFHxS: 0.058; PFHxS Ʃ branched isomers: 0.006; PFHpS: 0.037; n-PFOS: 0.114; PFOS Ʃ branched isomers: 0.122; PFNS: 0.019; PFDS: 0.010; PFECHS: 0.004; 4:2 FTSA: 0.009; 6:2 FTSA: 0.010; 8:2 FTSA: 0.019; HFPO-DA: 0.010; DONA: 0.009; cC6O4: 0.010; 6:2 Cl-PFESA: 0.009; and 8:2 Cl-PFESA: 0.004 µg/L

## Discussion

In this work, a method for the determination of 30 PFASs in human plasma has been set up and fully validated. The target analytes were carefully chosen in order to include both legacy PFASs belonging to different chemical classes and emerging fluorinated compounds whose environmental diffusion could be on the rise.

The development of this analytical method presented some challenges. In order to delay the possible PFAS contaminations derived from the HPLC system, a trap column was installed before the autosampler compartment, as suggested by previous applications [42, 49–51, 71, 72]. It has been reported that PFASs, in particular those with a long perfluoroalkyl chain, if diluted in water, can be adsorbed by laboratory material such as polypropylene [73] or glass [74], while this effect is not expected in pure undiluted biological samples characterised by abundant matrix components, or in samples dissolved mainly in an organic solvent [75–77]. For these reasons, dilution of standard solutions in water were avoided and, in general, the numbers of subsampling steps were kept as low as possible to reduce possible losses and/or contaminations. Further verifications were conducted analysing procedural blanks, in order to assess any contribution to overall contamination from every step of the entire measurement procedure, from the blood sampling to the collection in autosampler vials with PTFE-septum caps. PFASs leaching from PTFE-containing labware are recognised as sources of interference during PFAS analyses [77]; indeed, during the method development, we also tested polypropylene (PP) caps. While eliminating the risk of PFAS contaminations, the use of PP caps determined a significant evaporation of methanol as the PP cap does not re-seal after needle puncture. To demonstrate the suitability of PTFE-containing caps, replicates of procedural blanks in contact with both materials (PP and PTFE caps) were analysed. No signal differences were recorded among the two preparations, as confirmed consistently in different analytical sequences. Therefore, we considered the use of PFTE-containing caps as an adequate analytical practice, as long as the monitoring of interferences is routinely conducted through the analysis of procedural blanks.

Unlike other PFASs, HFPO-DA presented a peculiar fragmentation behaviour as an in-source fragmentation was observed, as previously described [78]. In order to increase sensitivity, the most intense fragment generated was chosen for both the native compound and its related isotopic labelled internal standard, despite the latter not matching the quant. transition; however, we verified its suitability through the good results obtained within method validation.

The sample preparation involved the protein precipitation with an organic solvent, the centrifugation, and the injection of the supernatant onto the HPLC system. During method development, we took into consideration a further purification step with a solid-phase extraction (SPE) using weak anionic exchange (WAX) cartridges (Waters, Sesto San Giovanni, Italy) (data not shown). Indeed, WAX cartridge can be very useful for the analysis of strong acidic compounds as PFASs [79]. Nevertheless, after performing some experiments, we decided to avoid its use for some reasons: (1) a matrix effect test suggested that the usage of SPE did not improve the burden of the matrix effect for the analytes and that the additional manual steps required affected reproducibility; (2) considerable contamination with PFBA derived from WAX cartridges was observed, as declared by the producer [80]; and (3) one of the considered analytes is not a strong acidic compound (PFOSA), thus requiring the collection and analysis of the eluate derived from the cartridge washing step, in turn reducing the throughput of the assay. Only a few other methods analysed PFASs with a sample preparation consisting only of a protein precipitation without further cleaning [42–44, 81]. The main advantages are the few steps and short time required for sample preparation, the low amount of solvent used, and the lower cost for consumables.

The main strength of the present method is the quantitation of several emerging PFASs, with great sensitivity: LLOQs of the present work were lower for HFPO-DA, F-53B-related analytes, and DONA [16, 43, 44, 56] and comparable for PFECHS [43] by comparing them with those reported by published methods. To the best of our knowledge, this is the first method able to quantify cC6O4 in human blood matrices.

Another strength of the present method is that we used a pooled human plasma as a blank matrix: most of the previous method used similar animal blood matrices such as calf serum and plasma, as a surrogate matrix containing lower amounts of ubiquitous PFASs [48, 54, 56, 82]. We bought commercial human pooled plasma from Biowest (Nuaillé, France), but it was not suitable for the method because of the high levels of PFOA and PFOS. Therefore, we screened several real human samples and created a pooled plasma mixing only those containing low levels of analytes (“Blank matrix” section). The main advantage of this approach is that, unlike other methods which used a surrogate bovine matrix, the calibration curves were prepared in real human plasma, thus allowing working with an ideal control matrix, matching the matrix of the unknown samples. A limitation is represented by the higher LLOQs obtained for some compounds, especially those of PFOA, n-PFHxS, n-PFOS, and PFOS ∑ branched isomers, which were still adequate for the quantitation of these compounds at the levels usually found in the general population (Table 3). LLOQs for all other compounds were considerably low and often lower than most of the others previously reported [83, 84].

An additional strength of this work is the extensive validation of the method, which was precise and accurate as shown by results collected over the course of 8 months (Table 2 and S5). The external verification of the method, even though performed only on a limited number of analytes, is a confirmation of its accuracy and robustness. The external verification also confirmed the stability of those PFASs in serum samples, as they were analysed at least 1 year after being prepared for the HBM4EU project, yielding accurate results. Furthermore, although our method was developed in plasma, the results obtained suggest the applicability of the method also on serum matrix for the analytes included in the ICI-Equas and the G-Equas. Therefore, the presence or absence of clotting factors in the matrix does not affect the capability of our method to properly quantify the considered analytes. Finally, another advantage of this method is the small amount of human plasma required to analyse a sample (20 µL).

Regarding PFHxS and PFOS, we were also able to separate the linear isomer from all the branched-chain isomers and quantified the latter as a sum of branched isomers by referring to its certified concentration. The importance of assessing human exposure to PFAS isomers has been reported [85].

The application of the method was intended to verify its performance and was applied only to a small subset of samples. As expected, PFOA, PFOS, PFHxS, and other long-chain PFAAs (PFNA, PFDA, PFUnDA, PFTrDA, PFHpS) were detected in most samples also showing the highest median concentrations. Among the emerging compounds, PFECHS were always found, while PFEAs as HFPO-DA, DONA, cC6O4, and F-53B-related analytes were mostly not quantifiable.

In conclusion, the present analytical method is a suitable tool for the biological monitoring of both traditional and emerging PFASs, for which human exposure may be on the rise; further studies are thus required to monitor their presence in larger populations and to assess their toxicokinetics and toxicological properties.

## Supplementary Information

Below is the link to the electronic supplementary material.

Supplementary file1 (DOCX 72 kb)

## Data Availability

All data and material are available upon request to the corresponding author.

## Abbreviations

11Cl-PF3OUdS: Commercial solution containing the 8:2 Cl-PFESA standard
4:2 FTSA: 1H,1H,2H,2H-Perfluorohexanesulfonic acid
6:2 Cl-PFESA: 2-(6-Chloro-1,1,2,2,3,3,4,4,5,5,6,6-dodecafluorohexoxy)-1,1,2,2-tetrafluoroethanesulfonic acid
6:2 FTSA: 1H,1H,2H,2H-Perfluorooctanesulfonic acid
8:2 Cl-PFESA: 2-(8-Chloro-1,1,2,2,3,3,4,4,5,5,6,6,7,7,8,8-hexadecafluorooctoxy)-1,1,2,2-tetrafluoroethanesulfonic acid
8:2 FTSA: 1H,1H,2H,2H-Perfluorodecanesulfonic acid
9Cl-PF3ONS: Commercial solution containing the 6:2 Cl-PFESA standard
ARPAV: Regional Agency for Environmental Protection and Prevention of Veneto
ARPA Lombardia: Regional Agency for Environmental Protection of Lombardy
CAS: Chemical Abstracts Service
cC6O4: (cis/trans)-Perfluoro([5-methoxy-1,3-dioxolan-4-yl]oxy)acetic acid
CE: Collision energies
d3-N-MeFOSAA: N-Methyl-d3-perfluoro-1-octanesulfonamidoacetic acid
d5-N-EtFOSAA: N-Ethyl-d5-perfluoro-1-octanesulfonamidoacetic acid
DF: Dilution factor
DONA: 3-H-Perfluoro-4,8-dioxanonanoic acid
EDTA: Ethylenediaminetetraacetic acid
EMA: European Medicines Agency
FDA: Food and Drug Administration
G-EQUAS: German External Quality Assessment Scheme
HFPO-DA: 2,3,3,3-Tetrafluoro-2-(heptafluoropropoxy)propanoic acid
ICH: International Council for Harmonisation of Technical Requirements for Pharmaceuticals for Human Use
ICI-EQUAS: Interlaboratory Comparison Investigations and External Quality Assurance Schemes
IS: Internal standards
LC–ESI–MS/MS: Liquid chromatography-electrospray ionisation tandem mass spectrometry
LLOQ: Lower limit of quantitation
M2-4:2 FTS: Sodium 1H,1H,2H,2H-perfluoro-1-[1,2-13C2]hexanesulfonate
M2-6:2 FTS: Sodium 1H,1H,2H,2H-perfluoro-1-[1,2-13C2]octanesulfonate
M2-8:2 FTS: Sodium 1H,1H,2H,2H-perfluoro-1-[1,2-13C2]decanesulfonate
M2PFDoA: Perfluoro-n-[1,2-13C2]dodecanoic acid
M2PFTeDA: Perfluoro-n-[1,2-13C2]tetradecanoic acid
M3HFPO-DA: 2,3,3,3-Tetrafluoro-2-(1,1,2,2,3,3,3-heptafluoropropoxy)-13C3-propanoic acid
M3PFBS: Sodium perfluoro-1-[2,3,4-13C3]butanesulfonate
M3PFHxS: Sodium perfluoro-1-[1,2,3-13C3]hexanesulfonate
M4PFBA: Perfluoro-n-[13C4]butanoic acid
M4PFHpA: Perfluoro-n-[1,2,3,4-13C4]heptanoic acid
M5PFHxA: Perfluoro-n-[1,2,3,4,6-13C5]hexanoic acid
M5PFPeA: Perfluoro-n-[13C5]pentanoic acid
M6PFDA: Perfluoro-n-[1,2,3,4,5,6-13C6]decanoic acid
M7PFUdA: Perfluoro-n-[1,2,3,4,5,6,7-13C7]undecanoic acid
M8FOSA: Perfluoro-1-[13C8]octanesulfonamide
M8PFOA: Perfluoro-n-[13C8]octanoic acid
M8PFOS: Sodium perfluoro-1-[13C8]octanesulfonate
M9PFNA: Perfluoro-n-[13C9]nonanoic acid
NaDONA: Commercial solution containing the DONA standard
N-EtFOSAA: N-Ethylperfluorooctane sulfonamidoacetic acid
N-MeFOSAA: N-Methylperfluorooctane sulfonamidoacetic acid
P5MeODIOXOAc: Commercial solution containing the cC6O4 standard
PFAAs: Perfluoroalkyl acids
PFAC-24PAR: Commercial solution containing the 24 native standards
PFASs: Per-/polyfluoroalkyl substances
PFBA: Perfluorobutanoic acid
PFBS: Perfluorobutanesulfonic acid
PFCAs: Perfluoroalkylcarboxylic acids
PFDA: Perfluorodecanoid acid
PFDoDA: Perfluorododecanoic acid
PFDS: Perfluorodecane sulfonic acid
PFEAs: Per-/polyfluoroalkyl ether acids
PFECAs: Per-/polyfluoroalkyl ether carboxylic acids
PFECHS: 1,2,2,3,3,4,5,5,6,6-Decafluoro-4-(1,1,2,2,2-pentafluoroethyl)cyclohexane-1-sulfonic acid
PFESAs: Per-/polyfluoroalkyl ether sulfonic acids
PFHpA: Perfluoroheptanoic acid
PFHpS: Perfluoroheptanesulfonic acid
PFHxA: Perfluorohexanoic acid
PFHxS: Perfluorohexanesulfonic acid
PFNA: Perfluorononanoic acid
PFNS: Perfluorononanesulfonic acid
PFOA: Perfluorooctanoic acid
PFOS: Perfluorooctanesulfonic acid
PFOSA: Perfluorooctanesulfonamide
PFPeA: Perfluoropentanoic acid
PFPeS: Perfluoropentanesulfonic acid
PFSAs: Perfluoroalkylsulfonic acids
PFTeDA: Perfluorotetradecanoic acid
PFTrDA: Perfluorotridecanoic acid
PFUnDA: Perfluoroundecanoic acid
POP: Persistent organic pollutant
POSF: Perfluorooctanesulfonyl fluoride
PP: Polypropylene
PTFE: Polytetrafluoroethylene
QC: Quality control solution
Qual.: Qualifier transition
Quant.: Quantifier transition
REACh: Registration, Evaluation, Authorisation and Restriction of Chemicals
RSD: Relative standard deviation
RT: Retention time
SIBioC: Italian Society of Clinical Biochemistry and Clinical Molecular Biology
s-MRM: Scheduled multiple reaction monitoring scan type
SPE: Solid-phase extraction
ULOQ: Upper limit of quantitation
WAX: Weak anionic exchange
