# Correction to: Development and validation of an LC–MS/MS method for the quantitation of 30 legacy and emerging per- and polyfluoroalkyl substances (PFASs) in human plasma, including HFPO‑DA, DONA, and cC6O4

**DOI:** 10.1007/s00216-022-03873-3

**Published:** 2022-01-14

**Authors:** Gianfranco Frigerio, Simone Cafagna, Elisa Polledri, Rosa Mercadante, Silvia Fustinoni

**Affiliations:** 1 Department of Clinical Sciences and Community Health, University of Milan, via S. Barnaba, 8, 20122 Milan, Italy; 2 Occupational Health Unit, Fondazione IRCCS Ca’ Granda Ospedale Maggiore Policlinico, Milan, Italy

**Correction to** **:** **Analytical and Bioanalytical Chemistry (2022) 414:1259–1278**

**https://doi.org/10.1007/s00216-021-03762-1**

The article “Development and validation of an LC–MS/MS method for the quantitation of 30 legacy and emerging per- and polyfluoroalkyl substances (PFASs) in human plasma, including HFPO‑DA, DONA, and cC6O4”, written by Gianfranco Frigerio, Simone Cafagna, Elisa Polledri, Rosa Mercadante and Silvia Fustinoni, was originally published Online First without Open Access. After publication in volume 414, issue 3, page 1259–1278 the author decided to opt for Open Choice and to make the article an Open Access publication. Therefore, the copyright of the article has been changed to © The Author(s) 2022 and the article is forthwith distributed under the terms of the Creative Commons Attribution 4.0 International License, which permits use, sharing, adaptation, distribution and reproduction in any medium or format, as long as you give appropriate credit to the original author(s) and the source, provide a link to the Creative Commons licence, and indicate if changes were made. The images or other third party material in this article are included in the article’s Creative Commons licence, unless indicated otherwise in a credit line to the material. If material is not included in the article’s Creative Commons licence and your intended use is not permitted by statutory regulation or exceeds the permitted use, you will need to obtain permission directly from the copyright holder. To view a copy of this licence, visit http://creativecommons.org/licenses/by/4.0.

