# Supplementary material for: Development and validation of an LC–MS/MS method for the quantitation of 30 legacy and emerging per- and polyfluoroalkyl substances (PFASs) in human plasma, including HFPO-DA, DONA, and cC6O4
Source: Anal Bioanal Chem. 2021 Dec 15;414(3):1259–78. doi: 10.1007/s00216-021-03762-1 (PMC8760233; doi:10.1007/s00216-021-03762-1)
Supplement: Supplementary file 1 — Supplementary file1 (DOCX 72 kb) [file 216_2021_3762_MOESM1_ESM.docx]

**Development and validation of an LC-MS/MS method for the quantitation of 30 legacy and emerging per- and polyfluoroalkyl substances (PFASs) in human plasma, including HFPO-DA, DONA, and cC6O4**

Gianfranco Frigerio, Simone Cafagna, Elisa Polledri, Rosa Mercadante, Silvia Fustinoni

**Supplementary material**

**Table S1** Components of each native standard stock solution/mixture, along with relative specifications.

| **Stock solution/mixture** | **Native standard** | **Abbreviation** | **CAS** | **Chemical purity (%)** | **Concentration (µg/L) ± 5%** |
| --- | --- | --- | --- | --- | --- |
| PFAC-24PAR | Perfluorobutanoic acid | PFBA | 375-22-4 | >98 | 2.00*10^3^ |
| PFAC-24PAR | Perfluoropentanoic acid | PFPeA | 2706-90-3 | >98 | 2.00*10^3^ |
| PFAC-24PAR | Perfluorohexanoic acid | PFHxA | 307-24-4 | >98 | 2.00*10^3^ |
| PFAC-24PAR | Perfluoroheptanoic acid | PFHpA | 375-85-9 | >98 | 2.00*10^3^ |
| PFAC-24PAR | Perfluorooctanoic acid | PFOA | 335-67-1 | >98 | 2.00*10^3^ |
| PFAC-24PAR | Perfluorononanoic acid | PFNA | 375-95-1 | >98 | 2.00*10^3^ |
| PFAC-24PAR | Perfluorodecanoid acid | PFDA | 335-76-2 | >98 | 2.00*10^3^ |
| PFAC-24PAR | Perfluoroundecanoic acid | PFUnDA | 2058-94-8 | >98 | 2.00*10^3^ |
| PFAC-24PAR | Perfluorododecanoic acid | PFDoDA | 307-55-1 | >98 | 2.00*10^3^ |
| PFAC-24PAR | Perfluorotridecanoic acid | PFTrDA | 72629-94-8 | >98 | 2.00*10^3^ |
| PFAC-24PAR | Perfluorotetradecanoic acid | PFTeDA | 376-06-7 | >98 | 2.00*10^3^ |
| PFAC-24PAR | Perfluorooctanesulfonamide | PFOSA | 754-91-6 | >98 | 2.00*10^3^ |
| PFAC-24PAR | N-Methylperfluorooctane sulfonamidoacetic acid | N-MeFOSAA | 2355-31-9 | >98 | 2.00*10^3^ |
| PFAC-24PAR | N-Ethylperfluorooctane sulfonamidoacetic acid | N-EtFOSAA | 2991-50-6 | >98 | 2.00*10^3^ |
| PFAC-24PAR | Perfluorobutanesulfonic acid | PFBS | 375-73-5 | >98 | 1.78*10^3^ |
| PFAC-24PAR | Perfluoropentanesulfonic acid | PFPeS | 2706-91-4 | >98 | 1.89*10^3^ |
| PFAC-24PAR | Perfluorohexanesulfonic acid: linear isomer | PFHxS | 355-46-4 | >98 | 1.48*10^3^ |
| PFAC-24PAR | Perfluorohexanesulfonic acid: Ʃ branched isomers |  |  |  | 345 |
| PFAC-24PAR | Perfluoroheptanesulfonic acid | PFHpS | 375-92-8 | >98 | 1.90*10^3^ |
| PFAC-24PAR | Perfluorooctanesulfonic acid: linear isomer | PFOS | 1763-23-1 | >98 | 1.46*10^3^ |
| PFAC-24PAR | Perfluorooctanesulfonic acid: Ʃ branched isomers |  |  |  | 392 |
| PFAC-24PAR | Perfluorononanesulfonic acid | PFNS | 68259-12-1 | >98 | 1.92*10^3^ |
| PFAC-24PAR | Perfluorodecane sulfonic acid | PFDS | 335-77-3 | >98 | 1.93*10^3^ |
| PFAC-24PAR | 1H,1H,2H,2H-Perfluorohexanesulfonic acid | 4:2 FTSA | 757124-72-4 | >98 | 1.88*10^3^ |
| PFAC-24PAR | 1H,1H,2H,2H-Perfluorooctanesulfonic acid | 6:2 FTSA | 27619-97-2 | >98 | 1.90*10^3^ |
| PFAC-24PAR | 1H,1H,2H,2H-Perfluorodecanesulfonic acid | 8:2 FTSA | 39108-34-4 | >98 | 1.92*10^3^ |
| P5MeODIOXOAc | rac-(cis/trans)-Perfluoro([5-methoxy-1,3-dioxolan-4-yl]oxy)acetic acid | cC6O4 | 1190931-41-9 | >98 | 50.0*10^3^ |
| NaDONA | 3-H-perfluoro-4,8-dioxanonanoic acid | DONA | 919005-14-4 | >98 | 47.2*10^3^ |
| 9Cl-PF3ONS | 2-(6-Chloro-1,1,2,2,3,3,4,4,5,5,6,6-dodecafluorohexoxy)-1,1,2,2-tetrafluoroethanesulfonic acid | 6:2 Cl-PFESA | 756426-58-1 | >98 | 46.7*10^3^ |
| 11Cl-PF3OUdS | 2-(8-Chloro-1,1,2,2,3,3,4,4,5,5,6,6,7,7,8,8-hexadecafluorooctoxy)-1,1,2,2-tetrafluoroethanesulfonic acid | 8:2 Cl-PFESA | 763051-92-9 | >98 | 47.2*10^3^ |
| HFPO-DA | 2,3,3,3-Tetrafluoro-2-(heptafluoropropoxy)propanoic acid | HFPO-DA | 13252-13-6 | >98 | 50.0*10^3^ |
| PFECHS | (cis70%/trans30%)-Cyclohexanesulfonicacid, 1,2,2,3,3,4,5,5,6,6-decafluoro-4-(1,1,2,2,2-pentafluoroethyl)- | PFECHS | 646-83-3 | >98 | 46.2*10^3^ |

**Table S2** Components of each mass labelled standard stock solution/mixture, along with relative specifications.

| **Product code** | **Mass labelled standard** | **Abbreviation** | **Concentration (µg/L) ± 5 %** | **Chemical purity (%)** | **Isotopic purity (%)** |
| --- | --- | --- | --- | --- | --- |
| MPFAC-24ES | Perfluoro-n-[^13^C_4_]butanoic acid | M4PFBA | 1.00*10^3^ | >98 | ≥99 |
| MPFAC-24ES | Perfluoro-n-[^13^C_5_]pentanoic acid | M5PFPeA | 1.00*10^3^ | >98 | ≥99 |
| MPFAC-24ES | Perfluoro-n-[1,2,3,4,6-^13^C_5_]hexanoic acid | M5PFHxA | 1.00*10^3^ | >98 | ≥99 |
| MPFAC-24ES | Perfluoro-n-[1,2,3,4-^13^C_4_]heptanoic acid | M4PFHpA | 1.00*10^3^ | >98 | ≥99 |
| MPFAC-24ES | Perfluoro-n-[^13^C_8_]octanoic acid | M8PFOA | 1.00*10^3^ | >98 | ≥99 |
| MPFAC-24ES | Perfluoro-n-[^13^C_9_]nonanoic acid | M9PFNA | 1.00*10^3^ | >98 | ≥99 |
| MPFAC-24ES | Perfluoro-n-[1,2,3,4,5,6-^13^C_6_]decanoic acid | M6PFDA | 1.00*10^3^ | >98 | ≥99 |
| MPFAC-24ES | Perfluoro-n-[1,2,3,4,5,6,7-13C_7_]undecanoic acid | M7PFUdA | 1.00*10^3^ | >98 | ≥99 |
| MPFAC-24ES | Perfluoro-n-[1,2-^13^C_2_]dodecanoic acid | M2PFDoA | 1.00*10^3^ | >98 | ≥99 |
| MPFAC-24ES | Perfluoro-n-[1,2-^13^C_2_]tetradecanoic acid | M2PFTeDA | 1.00*10^3^ | >98 | ≥99 |
| MPFAC-24ES | Perfluoro-1-[^13^C_8_]octanesulfonamide | M8FOSA | 1.00*10^3^ | >98 | ≥99 |
| MPFAC-24ES | N-methyl-d_3_-perfluoro-1-octanesulfonamidoacetic acid | d3-N-MeFOSAA | 1.00*10^3^ | >98 | ≥98 |
| MPFAC-24ES | N-ethyl-d_5_-perfluoro-1-octanesulfonamidoacetic acid | d5-N-EtFOSAA | 1.00*10^3^ | >98 | ≥98 |
| MPFAC-24ES | Sodium perfluoro-1-[2,3,4-^13^C_3_]butanesulfonate | M3PFBS | 1.00*10^3^ | >98 | ≥99 |
| MPFAC-24ES | Sodium perfluoro-1-[1,2,3-^13^C_3_]hexanesulfonate | M3PFHxS | 1.00*10^3^ | >98 | ≥99 |
| MPFAC-24ES | Sodium perfluoro-1-[^13^C_8_]octanesulfonate | M8PFOS | 1.00*10^3^ | >98 | ≥99 |
| MPFAC-24ES | Sodium 1H,1H,2H,2H-perfluoro-1-[1,2-^13^C_2_]hexanesulfonate | M2-4:2 FTS | 1.00*10^3^ | >98 | ≥99 |
| MPFAC-24ES | Sodium 1H,1H,2H,2H-perfluoro-1-[1,2-^13^C_2_]octanesulfonate | M2-6:2 FTS | 1.00*10^3^ | >98 | ≥99 |
| MPFAC-24ES | Sodium 1H,1H,2H,2H-perfluoro-1-[1,2-^13^C_2_]decanesulfonate | M2-8:2 FTS | 1.00*10^3^ | >98 | ≥99 |
| M3HFPO-DA | 2,3,3,3-Tetrafluoro-2-(1,1,2,2,3,3,3-heptafluoropropoxy)-^13^C_3_-propanoic acid | M3HFPO-DA | 50.0*10^3^ | N/A | N/A |

N/A: not available

**Table S3** Concentrations of analytes for each calibration and QC level. The range of concentrations considered for the linear regression (from LLOQ to ULOQ) are highlighted.

|  | Concentrations of analytes in each standard (µg/L) | | | | | | | | | | | | | |
| --- | --- | --- | --- | --- | --- | --- | --- | --- | --- | --- | --- | --- | --- | --- |
| Analytes | level 1 | Level  2 | Level  3 | Level  4 | Level  5  (QC) | Level  6 | Level  7 | Level  8 (QC) | Level  9 | Level  10  (QC) | Level  11 | Level  12 | Level  13 | Level  14 |
| PFBA | 0.005 | 0.010 | 0.020 | 0.039 | 0.078 | 0.156 | 0.313 | 0.625 | 1.25 | 2.50 | 5.00 | 10.0 | 20.0 | 40.0 |
| PFPeA | 0.005 | 0.010 | 0.020 | 0.039 | 0.078 | 0.156 | 0.313 | 0.625 | 1.25 | 2.50 | 5.00 | 10.0 | 20.0 | 40.0 |
| PFHxA | 0.005 | 0.010 | 0.020 | 0.039 | 0.078 | 0.156 | 0.313 | 0.625 | 1.25 | 2.50 | 5.00 | 10.0 | 20.0 | 40.0 |
| PFHpA | 0.005 | 0.010 | 0.020 | 0.039 | 0.078 | 0.156 | 0.313 | 0.625 | 1.25 | 2.50 | 5.00 | 10.0 | 20.0 | 40.0 |
| PFOA | 0.005 | 0.010 | 0.020 | 0.039 | 0.078 | 0.156 | 0.313 | 0.625 | 1.25 | 2.50 | 5.00 | 10.0 | 20.0 | 40.0 |
| PFNA | 0.005 | 0.010 | 0.020 | 0.039 | 0.078 | 0.156 | 0.313 | 0.625 | 1.25 | 2.50 | 5.00 | 10.0 | 20.0 | 40.0 |
| PFDA | 0.005 | 0.010 | 0.020 | 0.039 | 0.078 | 0.156 | 0.313 | 0.625 | 1.25 | 2.50 | 5.00 | 10.0 | 20.0 | 40.0 |
| PFUnDA | 0.005 | 0.010 | 0.020 | 0.039 | 0.078 | 0.156 | 0.313 | 0.625 | 1.25 | 2.50 | 5.00 | 10.0 | 20.0 | 40.0 |
| PFDoDA | 0.005 | 0.010 | 0.020 | 0.039 | 0.078 | 0.156 | 0.313 | 0.625 | 1.25 | 2.50 | 5.00 | 10.0 | 20.0 | 40.0 |
| PFTrDA | 0.005 | 0.010 | 0.020 | 0.039 | 0.078 | 0.156 | 0.313 | 0.625 | 1.25 | 2.50 | 5.00 | 10.0 | 20.0 | 40.0 |
| PFTeDA | 0.005 | 0.010 | 0.020 | 0.039 | 0.078 | 0.156 | 0.313 | 0.625 | 1.25 | 2.50 | 5.00 | 10.0 | 20.0 | 40.0 |
| PFOSA | 0.005 | 0.010 | 0.020 | 0.039 | 0.078 | 0.156 | 0.313 | 0.625 | 1.25 | 2.50 | 5.00 | 10.0 | 20.0 | 40.0 |
| N-MeFOSAA | 0.005 | 0.010 | 0.020 | 0.039 | 0.078 | 0.156 | 0.313 | 0.625 | 1.25 | 2.50 | 5.00 | 10.0 | 20.0 | 40.0 |
| N-EtFOSAA | 0.005 | 0.010 | 0.020 | 0.039 | 0.078 | 0.156 | 0.313 | 0.625 | 1.25 | 2.50 | 5.00 | 10.0 | 20.0 | 40.0 |
| PFBS | 0.004 | 0.009 | 0.017 | 0.035 | 0.069 | 0.139 | 0.277 | 0.555 | 1.11 | 2.22 | 4.44 | 8.88 | 17.8 | 35.5 |
| PFPeS | 0.005 | 0.009 | 0.018 | 0.037 | 0.074 | 0.147 | 0.295 | 0.589 | 1.18 | 2.36 | 4.71 | 9.43 | 18.9 | 37.7 |
| n-PFHxS | 0.004 | 0.007 | 0.014 | 0.029 | 0.058 | 0.116 | 0.232 | 0.464 | 0.927 | 1.85 | 3.71 | 7.42 | 14.8 | 29.7 |
| PFHxS ∑ branched isomers | 0.001 | 0.002 | 0.003 | 0.007 | 0.013 | 0.027 | 0.054 | 0.108 | 0.216 | 0.431 | 0.862 | 1.72 | 3.45 | 6.90 |
| PFHpS | 0.005 | 0.009 | 0.019 | 0.037 | 0.074 | 0.149 | 0.298 | 0.595 | 1.19 | 2.38 | 4.76 | 9.52 | 19.0 | 38.1 |
| n-PFOS | 0.004 | 0.007 | 0.014 | 0.029 | 0.057 | 0.114 | 0.229 | 0.457 | 0.91 | 1.83 | 3.66 | 7.31 | 14.6 | 29.3 |
| PFOS ∑ branched isomers | 0.001 | 0.002 | 0.004 | 0.008 | 0.015 | 0.031 | 0.061 | 0.122 | 0.245 | 0.490 | 0.979 | 1.96 | 3.92 | 7.84 |
| PFNS | 0.005 | 0.009 | 0.019 | 0.038 | 0.075 | 0.150 | 0.301 | 0.601 | 1.20 | 2.40 | 4.81 | 9.62 | 19.2 | 38.5 |
| PFDS | 0.005 | 0.009 | 0.019 | 0.038 | 0.076 | 0.151 | 0.302 | 0.604 | 1.21 | 2.42 | 4.83 | 9.67 | 19.3 | 38.7 |
| PFECHS | 0.005 | 0.009 | 0.018 | 0.036 | 0.072 | 0.144 | 0.289 | 0.578 | 1.16 | 2.31 | 4.62 | 9.24 | 18.5 | 37.0 |
| 4:2 FTSA | 0.005 | 0.009 | 0.018 | 0.037 | 0.073 | 0.147 | 0.293 | 0.586 | 1.17 | 2.34 | 4.69 | 9.38 | 18.8 | 37.5 |
| 6:2 FTSA | 0.005 | 0.009 | 0.019 | 0.037 | 0.074 | 0.149 | 0.298 | 0.595 | 1.19 | 2.38 | 4.76 | 9.52 | 19.0 | 38.1 |
| 8:2 FTSA | 0.005 | 0.009 | 0.019 | 0.038 | 0.075 | 0.150 | 0.301 | 0.601 | 1.20 | 2.40 | 4.81 | 9.62 | 19.2 | 38.5 |
| HFPO-DA | 0.005 | 0.010 | 0.020 | 0.039 | 0.078 | 0.156 | 0.313 | 0.625 | 1.25 | 2.50 | 5.00 | 10.0 | 20.0 | 40.0 |
| DONA | 0.005 | 0.009 | 0.018 | 0.037 | 0.074 | 0.148 | 0.295 | 0.590 | 1.18 | 2.36 | 4.72 | 9.45 | 18.9 | 37.8 |
| cC6O4 | 0.005 | 0.010 | 0.020 | 0.039 | 0.078 | 0.156 | 0.313 | 0.625 | 1.25 | 2.50 | 5.00 | 10.0 | 20.0 | 40.0 |
| 6:2 Cl-PFESA | 0.005 | 0.009 | 0.018 | 0.036 | 0.073 | 0.146 | 0.292 | 0.584 | 1.17 | 2.33 | 4.67 | 9.34 | 18.7 | 37.4 |
| 8:2 Cl-PFESA | 0.005 | 0.009 | 0.018 | 0.037 | 0.074 | 0.148 | 0.295 | 0.590 | 1.18 | 2.36 | 4.72 | 9.44 | 18.9 | 37.8 |

**Table S4** Concentrations of the analytes quantified in the blank matrix, i.e. the pooled plasma obtained mixing human plasma samples containing relatively low levels of the analytes.

| **Analyte** | **Concentration in blank matrix (µg/L)** |
| --- | --- |
| PFBA | < LLOQ |
| PFPeA | < LLOQ |
| PFHxA | < LLOQ |
| PFHpA | < LLOQ |
| PFOA | 1.036 |
| PFNA | 0.254 |
| PFDA | 0.121 |
| PFUnDA | 0.080 |
| PFDoDA | < LLOQ |
| PFTrDA | 0.022 |
| PFTeDA | < LLOQ |
| PFOSA | < LLOQ |
| N-MeFOSAA | < LLOQ |
| N-EtFOSAA | < LLOQ |
| PFBS | < LLOQ |
| PFPeS | 0.020 |
| n-PFHxS | 0.379 |
| ∑PFHxS branched isomers | 0.023 |
| PFHpS | < LLOQ |
| n-PFOS | 1.129 |
| ∑PFOS branched isomers | 0.790 |
| PFNS | < LLOQ |
| PFDS | < LLOQ |
| PFECHS | 0.040 |
| 6:2 FTSA | < LLOQ |
| 8:2 FTSA | < LLOQ |
| 4:2 FTSA | < LLOQ |
| HFPO-DA | < LLOQ |
| DONA | < LLOQ |
| cC6O4 | < LLOQ |
| 6:2 Cl-PFESA | 0.018 |
| 8:2 Cl-PFESA | <LLOQ |

**Table S5** Results of the analyses of ICI-Equas samples. We did not participate in the exercise, but we used the samples as reference material and compared our results with those reported in the HBM4EU reports. All the samples from all rounds were analysed at the same time and again after 6 months. Within each analytical run the samples were analysed twice. Results are reported as accuracy (%theoretical) and Z-score obtained from two analytical batches; mean accuracy and mean Z-score (calculated as the mean of absolute values) are also shown.

|  | **Reference value** | **Accuracy**  **1° analysis** | **Accuracy**  **6 months later** | **Mean accuracy** | **Z-score,**  **1° analysis** | **Z-score,**  **6 months later** | **Mean**  **Z-score** |
| --- | --- | --- | --- | --- | --- | --- | --- |
| **Round 02** | | | | | | | |
| PFPeA | 0.189 ng/ml | 102.5 | 108.0 | 105.2 | 0.1 | 0.3 | 0.2 |
|  | 0.234 ng/ml | 105.3 | 116.0 | 110.6 | 0.2 | 0.6 | 0.4 |
| PFHxA | 0.323 ng/ml | 93.4 | 92.5 | 92.9 | -0.3 | -0.3 | 0.3 |
|  | 0.509 ng/ml | 79.7 | 90.6 | 85.2 | -0.8 | -0.4 | 0.6 |
| PFHpA | 0.396 ng/ml | 96.2 | 110.7 | 103.4 | -0.2 | 0.4 | 0.3 |
|  | 1.042 ng/ml | 86.0 | 111.9 | 99.0 | -0.6 | 0.5 | 0.5 |
| PFOA | 2.093 ng/ml | 97.4 | 107.0 | 102.2 | -0.1 | 0.3 | 0.2 |
|  | 3.649 ng/ml | 99.8 | 95.9 | 97.8 | 0.0 | -0.2 | 0.1 |
| PFNA | 0.577 ng/ml | 101.8 | 103.3 | 102.6 | 0.1 | 0.1 | 0.1 |
|  | 1.070 ng/ml | 96.3 | 107.2 | 101.8 | -0.1 | 0.3 | 0.2 |
| PFDA | 0.507 ng/ml | 95.0 | 106.6 | 100.8 | -0.2 | 0.3 | 0.2 |
|  | 0.946 ng/ml | 94.6 | 109.3 | 101.9 | -0.2 | 0.4 | 0.3 |
| PFUnDA | 0.331 ng/ml | 98.2 | 117.6 | 107.9 | -0.1 | 0.7 | 0.4 |
|  | 0.563 ng/ml | 92.9 | 108.9 | 100.9 | -0.3 | 0.4 | 0.3 |
| PFDoDA | 0.207 ng/ml | 91.4 | 110.1 | 100.7 | -0.3 | 0.4 | 0.4 |
|  | 0.333 ng/ml | 93.9 | 117.9 | 105.9 | -0.2 | 0.7 | 0.5 |
| PFBS | 0.159 ng/ml | 87.9 | 98.4 | 93.1 | -0.5 | -0.1 | 0.3 |
|  | 0.309 ng/ml | 83.1 | 96.6 | 89.9 | -0.7 | -0.1 | 0.4 |
| PFHxS | 0.725 ng/ml | 81.4 | 97.0 | 89.2 | -0.7 | -0.1 | 0.4 |
|  | 1.33 ng/ml | 82.0 | 99.9 | 90.9 | -0.7 | 0.0 | 0.4 |
| PFHpS | 0.210 ng/ml | 91.6 | 115.2 | 103.4 | -0.3 | 0.6 | 0.5 |
|  | 0.422 ng/ml | 86.4 | 111.8 | 99.1 | -0.5 | 0.5 | 0.5 |
| PFOS | 2.995 ng/ml | 99.9 | 107.2 | 103.5 | 0.0 | 0.3 | 0.1 |
|  | 4.672 ng/ml | 100.5 | 103.5 | 102.0 | 0.0 | 0.1 | 0.1 |
| **Round 03** | | | | | | | |
| PFPeA | 0.248 ng/mL | 90.7 | 99.7 | 95.2 | -0.4 | 0.0 | 0.2 |
|  | 0.306 ng/mL | 92.3 | 102.3 | 97.3 | -0.3 | 0.1 | 0.2 |
| PFHxA | 0.184 ng/mL | 93.1 | 80.1 | 86.6 | -0.3 | -0.8 | 0.5 |
|  | 0.308 ng/mL | 78.9 | 85.3 | 82.1 | -0.8 | -0.6 | 0.7 |
| PFHpA | 0.244 ng/mL | 90.8 | 101.4 | 96.1 | -0.4 | 0.1 | 0.2 |
|  | 0.588 ng/mL | 94.7 | 105.2 | 100.0 | -0.2 | 0.2 | 0.2 |
| PFOA | 0.945 ng/mL | 106.1 | 109.5 | 107.8 | 0.2 | 0.4 | 0.3 |
|  | 5.100 ng/mL | 102.8 | 97.7 | 100.3 | 0.1 | -0.1 | 0.1 |
| PFNA | 0.424 ng/mL | 91.2 | 99.0 | 95.1 | -0.4 | 0.0 | 0.2 |
|  | 0.817 ng/mL | 94.7 | 111.6 | 103.1 | -0.2 | 0.5 | 0.3 |
| PFDA | 0.369 ng/mL | 97.9 | 105.6 | 101.7 | -0.1 | 0.2 | 0.2 |
|  | 0.719 ng/mL | 93.6 | 114.0 | 103.8 | -0.3 | 0.6 | 0.4 |
| PFUnDA | 0.272 ng/mL | 97.6 | 110.3 | 103.9 | -0.1 | 0.4 | 0.3 |
|  | 0.497 ng/mL | 94.2 | 115.7 | 105.0 | -0.2 | 0.6 | 0.4 |
| PFDoDA | 0.151 ng/mL | 100.5 | 109.1 | 104.8 | 0.0 | 0.4 | 0.2 |
|  | 0.263 ng/mL | 92.6 | 119.1 | 105.9 | -0.3 | 0.8 | 0.5 |
| PFBS | 0.208 ng/mL | 84.4 | 94.8 | 89.6 | -0.6 | -0.2 | 0.4 |
|  | 0.396 ng/mL | 85.7 | 98.7 | 92.2 | -0.6 | -0.1 | 0.3 |
| PFHxS | 0.482 ng/mL | 77.0 | 90.1 | 83.6 | -0.9 | -0.4 | 0.7 |
|  | 0.814 ng/mL | 80.0 | 95.2 | 87.6 | -0.8 | -0.2 | 0.5 |
| PFHpS | 0.138 ng/mL | 104.4 | 118.1 | 111.3 | 0.2 | 0.7 | 0.5 |
|  | 0.271 ng/mL | 96.3 | 100.6 | 98.5 | -0.1 | 0.0 | 0.1 |
| PFOS | 1.672 ng/mL | 108.5 | 102.4 | 105.4 | 0.3 | 0.1 | 0.2 |
|  | 5.304 ng/mL | 98.1 | 106.0 | 102.0 | -0.1 | 0.2 | 0.2 |
| **Round 04** | | | | | | | |
| PFPeA | 0.396 ng/mL | 111.7 | 125.3 | 118.5 | 0.5 | 1.0 | 0.7 |
|  | 0.477 ng/mL | 114.8 | 121.1 | 117.9 | 0.6 | 0.8 | 0.7 |
| PFHxA | 0.133 ng/mL | 89.3 | 85.3 | 87.3 | -0.4 | -0.6 | 0.5 |
|  | 0.175 ng/mL | 94.9 | 82.1 | 88.5 | -0.2 | -0.7 | 0.5 |
| PFHpA | 0.189 ng/mL | 107.5 | 112.7 | 110.1 | 0.3 | 0.5 | 0.4 |
|  | 0.731 ng/mL | 104.4 | 102.1 | 103.3 | 0.2 | 0.1 | 0.1 |
| PFOA | 0.434 ng/mL | 101.8 | 112.9 | 107.4 | 0.1 | 0.5 | 0.3 |
|  | 11.444 ng/mL | 111.6 | 97.9 | 104.8 | 0.5 | -0.1 | 0.3 |
| PFNA | 0.221 ng/mL | 91.9 | 95.4 | 93.7 | -0.3 | -0.2 | 0.3 |
|  | 0.473 ng/mL | 93.0 | 101.0 | 97.0 | -0.3 | 0.0 | 0.2 |
| PFDA | 0.500 ng/mL | 93.0 | 107.3 | 100.1 | -0.3 | 0.3 | 0.3 |
|  | 0.769 ng/mL | 93.9 | 107.1 | 100.5 | -0.2 | 0.3 | 0.3 |
| PFUnDA | 0.234 ng/mL | 106.7 | 113.6 | 110.2 | 0.3 | 0.5 | 0.4 |
|  | 0.661 ng/mL | 90.3 | 108.9 | 99.6 | -0.4 | 0.4 | 0.4 |
| PFDoDA | 0.114 ng/mL | 108.2 | 130.3 | 119.2 | 0.3 | 1.2 | 0.8 |
|  | 0.352 ng/mL | 101.4 | 111.0 | 106.2 | 0.1 | 0.4 | 0.2 |
| PFBS | 0.118 ng/mL | 89.9 | 105.4 | 97.6 | -0.4 | 0.2 | 0.3 |
|  | 0.457 ng/mL | 88.9 | 98.4 | 93.6 | -0.4 | -0.1 | 0.3 |
| PFHxS | 0.390 ng/mL | 80.8 | 97.0 | 88.9 | -0.8 | -0.1 | 0.4 |
|  | 2.006 ng/mL | 83.5 | 96.2 | 89.9 | -0.7 | -0.2 | 0.4 |
| PFHpS | 0.306 ng/mL | 91.7 | 103.7 | 97.7 | -0.3 | 0.1 | 0.2 |
|  | 1.150 ng/mL | 86.2 | 100.4 | 93.3 | -0.6 | 0.0 | 0.3 |
| PFOS | 1.415 ng/mL | 116.7 | 107.9 | 112.3 | 0.7 | 0.3 | 0.5 |
|  | 8.227 ng/mL | 92.1 | 100.6 | 96.4 | -0.3 | 0.0 | 0.2 |

**Table S6** Results of the external verification G-Equas, for round 67. Our values are compared to those obtained from reference laboratories. The evaluation was positive as our results were between the tolerance ranges.

| **Analyte** | **Our value**  **(µg/L)** | **ref. value**  **(µg/L)** | **Tolerance range**  **(µg/L)** | **Evaluation**  **(µg/L)** |
| --- | --- | --- | --- | --- |
| **PFOA – A value** | 2.52 | 2.5 | 1.99 - 3.01 | + |
| **PFOA – B value** | 44.89 | 44.71 | 38.41 - 51.01 | + |
| **PFOS – A value** | 3.16 | 3.74 | 2.93 - 4.55 | + |
| **PFOS – B value** | 18.11 | 20.83 | 17.20 - 24.46 | + |

**Figure S1** Results of the external verification G-Equas for round 67. The graphs show the position of our values (blue square) as compared to the reference values (green line) and to the limits of the tolerance range (red lines).
